# Postural effects on intraocular pressure and ocular perfusion pressure in patients with non-arteritic anterior ischemic optic neuropathy

**DOI:** 10.1186/s12886-017-0441-3

**Published:** 2017-04-20

**Authors:** Jee Myung Yang, Sang Woo Park, Yong Sok Ji, Jaeryung Kim, Chungkwon Yoo, Hwan Heo

**Affiliations:** 1 Department of Ophthalmology, Chonnam National University Medical School and Hospital, 42 Jebong-ro, Dong-gu, Gwangju, 501-757 Republic of Korea; 2 Graduate School of Medical Science and Engineering, Korea Advanced Institute of Science and Technology (KAIST), Daejeon, Republic of Korea; 3 Department of Ophthalmology, Samsung Medical Center, Sungkyunkwan University School of Medicine, Seoul, Republic of Korea; 4 Department of Ophthalmology, Korea University College of Medicine, Seoul, Republic of Korea

**Keywords:** Intraocular pressure, Lateral decubitus position, Non-arteritic ischemic optic neuropathy, Ocular perfusion pressure, Postural change

## Abstract

**Background:**

To investigate postural effects on intraocular pressure (IOP) and ocular perfusion pressure (OPP) in patients with non-arteritic ischemic optic neuropathy (NAION).

**Methods:**

IOP and blood pressure (BP) were measured in 20 patients with unilateral NAION 10 min after changing to each of the following positions sequentially: sitting, supine, right lateral decubitus position (LDP), supine, left LDP, and supine. IOP was measured using a rebound tonometer and OPP was calculated using formulas based on mean BP. The dependent LDP (DLDP) was defined as the position when the eye of interest (affected or unaffected eye) was placed on the dependent side in the LDP.

**Results:**

IOPs were significantly higher (*P* = 0.020) and OPPs were significantly lower (*P* = 0.041) in the affected eye compare with the unaffected eye, with the affected eye in DLDP. Compared with the mean IOP of the unaffected eyes, the mean IOP of the affected eyes increased significantly (+2.9 ± 4.4 versus +0.7 ± 3.1 mmHg, respectively; *P* = 0.003) and the mean OPP decreased significantly (−6.7 ± 9.4 versus −4.9 ± 8.0 mmHg, respectively; *P* = 0.022) after changing positions from supine to DLDP. In addition, changing position from supine to DLDP showed significantly larger absolute changes in IOP (4.13 ± 3.19 mmHg versus 2.51 ± 1.92 mmHg, respectively; *P* = 0.004) and OPP (9.86 ± 5.69 mmHg versus 7.50 ± 5.49 mmHg, respectively; *P* = 0.009) in the affected eye compared with the unaffected eye. In the affected eye, there was a significant positive correlation between absolute change in IOP and OPP when changing position from supine to DLDP (Rho = 0.512, *P* = 0.021).

**Conclusions:**

A postural change from supine to DLDP caused significant fluctuations in IOP and OPP of the affected eye, and may significantly increase IOP and decrease OPP. Posture-induced IOP changes may be a predisposing factor for NAION development.

**Electronic supplementary material:**

The online version of this article (doi:10.1186/s12886-017-0441-3) contains supplementary material, which is available to authorized users.

## Background

Non-arteritic anterior ischemic optic neuropathy (NAION) is one of the common causes of visual loss in the middle-aged and elderly populations. It is characterized by a sudden onset, painless visual loss associated with optic disc swelling and peripapillary hemorrhage [1]. Although it is widely assumed that NAION results from circulatory insufficiency of the optic nerve head (ONH), its pathogenesis remains highly controversial.

Among various factors, ocular perfusion pressure (OPP) is presumed to play an important role in NAION pathogenesis. An inverse relationship between intraocular pressure (IOP) and OPP in situations of impaired blood flow autoregulation has been demonstrated [1, 2]. Therefore, IOP fluctuations can have a considerable impact on ONH blood flow in patients with systemic and local disorders that disrupt ONH autoregulation [3, 4].

Although there are individual variations, it is well known that IOP generally changes according to body position, and that a postural change from sitting to supine or lateral decubitus position (LDP) induces significant IOP elevation [5–7]. IOP elevation in the recumbent posture can impair perfusion pressure that in turn can predispose ONH to ischemic insult in some susceptible individuals [8].

Despite extensive studies of postural-induced IOP and OPP fluctuations in glaucoma, relatively little attention has been paid to NAION [6, 7]. Since most cases of NAION develop during sleeping, it is important to focus on the events (i.e., changes of body position), and their effects on ONH blood flow, that occurs during sleeping [9, 10]. Previously, James et al. [11] studied the effect of posture on the IOP and pulsatile ocular blood flow in patients with NAION. However, their study did not consider LDP, a posture that patients can adopt for a considerable amount of time while sleeping.

The purpose of this study is to investigate postural effects on IOP and OPP in patients with NAION; in particular, we focused on LDP-induced IOP changes in affected eyes to determine whether this postural change can significantly influence ONH perfusion.

## Methods

### Study design

This prospective, observational study was approved by the Institutional Review Board of Chonnam National University Hospital. It was conducted in accordance with the Declaration of Helsinki. A written informed consent was obtained from all patients before study initiation. We enrolled 20 Asian patients who had been newly diagnosed with unilateral NAION at the Neuro-ophthalmology Service, Department of Ophthalmology, Chonnam National University Hospital.

### Non-arteritic anterior ischemic optic neuropathy diagnosis

NAION diagnosis was based on the following clinical findings: acute painless monocular visual loss, presence of relative afferent pupillary defect, sectorial or generalized optic disc edema, peripapillary hemorrhage, and visual field loss consistent with optic neuropathy. All patients underwent complete ophthalmological examinations (including fluorescein angiography), cardiovascular examinations, cerebrovascular examination with brain magnetic resonance imaging, and blood examinations (including erythrocyte sedimentation rate and C-reactive protein). Patients with pain on ocular movement, history of temporal arteritis, previous ocular surgery (including recent cataract operation), intracranial lesion, or other ocular disorders that could result in visual defects were excluded. In addition, patients who had been previously diagnosed as glaucoma or those with history of ocular hypotensive therapy were excluded.

### Study measurements

All patients were admitted to the Neuro-ophthalmology Department of Chonnam National University Hospital, and IOP measurements were obtained between 6 pm and 8 pm. After administration of a local anesthetic (0.5% proparacaine hydrochloride), IOP measurements in the different positions were obtained using the Icare rebound tonometer (Icare PRO, Icare Finland Oy, Helsinki, Finland) by a single examiner (J.M.Y). IOP was measured after each posture had been maintained for 10 min in the following order: sitting (T1), supine (T2), right LDP (T3), supine (T4), left LDP (T5), and supine (T6) [12]. The dependent LDP (DLDP) was defined as the position when the eye of interest (affected or unaffected) was placed on the dependent (lower) side in LDP. To maintain the head position parallel to the bed, a soft pillow was placed under the head and neck when the patients were in the supine position and LDP. Three consecutive sets of measurements were taken for each position. The Icare rebound tonometer automatically averaged the IOP of the six measurements of each set and only values within normal variation between measurements were included. For data analysis, the mean values of three consecutive average values in each position were used.

Systolic and diastolic blood pressures (BP) were measured in each position at the heart level [13]. To calculate OPP, mean arterial pressure (MAP) was calculated according to the following equation: MAP = ([2 x diastolic BP] + systolic BP)/3. OPP was calculated in the sitting and lying positions using the formulas proposed by previous studies [7, 13, 14]:

1 $$ \mathrm{OPP}\ \mathrm{in}\ \mathrm{sitting}\ \mathrm{position}=95/140\ \mathrm{x}\ \mathrm{MAP}-\mathrm{IOP} $$

2 $$ \mathrm{OPP}\ \mathrm{in}\ \mathrm{lying}\ \mathrm{position}=115/130\ \mathrm{x}\ \mathrm{MAP}-\mathrm{IOP} $$

### Statistical analysis

All statistical analyses were performed using the SPSS software version 18.0 (IBM Corp., Armonk, NY, USA). A sample size calculation determined that 20 patients would be required to detect an anticipated IOP difference of 2.0 mmHg, at a standard deviation of 3.0 mmHg, with a power of 80% [7]. Detection of outliers was based on a criterion using z-score, and excluded from the data analysis [15] Wilcoxon-signed rank test was used to assess differences in continuous variables. Categorical variables were described as percentages and compared using the Chi-square test. The correlation between the changes of IOP and OPP values were analyzed by Spearman’s correlation method. *P*-values <0.05 indicated statistical significance.

## Results

The clinical characteristics of the patients are summarized in Table 1. Mean patient age was 59.60 ± 11.89 years (range: 42-84 years). Among the 20 patients, 8 (40%) were men and 12 were women (60%). Thirteen patients developed NAION in the right eye (65%) and seven in the left eye (35%). In the unaffected eyes, optic discs showed small vertical cup-to-disc ratios (0.18 ± 0.14). Mean duration from symptom onset was 8.35 ± 5.40 days (range, 0-21 days). All eyes had open angles on static gonioscopy.

**Table 1:** Clinical Characteristics of the Patients with Non-arteritic Ischemic Optic Neuropathy

General data	
Number of patients	20
Age, years	59.60 ± 11.89
Laterality, n (%)	
Right	13 (65)
Left	7 (35)
Sex, n (%)	
Male	8 (40)
Female	12 (60)
Initial BCVA, n (%)	
LP to HM	1 (5)
Count fingers to 20/200	6 (30)
20/160-20/50	4 (20)
≥ 20/40	9 (45)
Average CDR of fellow eye^a^	0.20 ± 0.15
Vertical CDR of fellow eye^b^	0.18 ± 0.14
SE, D	0.13 ± 1.24
Visual field - Initial MD, dB	−13.00 ± 8.80
Underlying systemic disease, n (%)	
Hypertension	5 (25)
Diabetes	4 (20)
Ischemic heart disease	2 (10)
Cerebrovascular disease	5 (25)
Thyroid disease	3 (15)
Hyperlipidemia	2 (10)
No major systemic disease	4 (20)

*BCVA* best-corrected visual acuity, *CDR* cup-to-disc ratio, *D* diopters, *HM* hand motion, *IOP* intraocular pressure, *LP* light perception, *MD* mean deviation, *SE* spherical equivalent

^a^The average CDR is the square root of the ratio of the area of the cup to the area of the disc

^b^The vertical CDR is the ratio of vertical cup diameter to vertical disc diameter

Table 2 lists the IOPs and calculated OPPs obtained in different body positions. Blood pressure data in different body positions are detailed in Additional file 1. Significant changes in IOP and OPP were observed in every changes of body positions from one to another in both affected and unaffected eyes (*P* value not shown), however, the mean IOP and OPP were not significantly different between eyes in either position (*P* > 0.05). Interestingly, IOPs were significantly higher (*P* = 0.020) and OPP were significantly lower (*P* = 0.041) in the affected eye compare with the unaffected eye, when values were determined with the affected eye in DLDP.

**Table 2:** Intraocular Pressure and Ocular Perfusion Pressure in the Different Body Position

	IOP (mmHg)			OPP (mmHg)		
	Affected Eye	Unaffected Eye	*P* value*	Affected Eye	Unaffected Eye	*P* value*
T1	16.6 ± 3.4 (10.5–23.3)	15.6 ± 2.9 (9.5–20.6)	0.055	50.1 ± 9.9 (35.4–77.6)	51.1 ± 11.0 (33.4–78.3)	0.055
T2	17.8 ± 3.5 (11.6–28.1)	17.4 ± 3.2 (11.8–24.1)	0.327	65.7 ± 12.4 (47.6–92.2)	66.2 ± 12.5 (46.9–93.7)	0.139
T3	20.2 ± 4.2 (13.8–29.9)	19.2 ± 3.7 (14.6–28.1)	0.457	59.8 ± 13.8 (37.3–83.2)	60.2 ± 14.0 (35.5–83.0)	0.355
T4	18.2 ± 2.7 (11.9–23.4)	17.7 ± 3.2 (12.6–25.5)	0.089	64.7 ± 13.1 (39.9–92.5)	65.8 ± 12.5 (46.8–92.1)	0.089
T5	20.3 ± 5.6 (14.0–32.2)	18.9 ± 4.7 (10.8–30.4)	0.287	53.1 ± 12.5 (38.4–81.0)	54.5 ± 13.5 (34.5–82.2)	0.422
T6	18.0 ± 3.6 (9.7–23.4)	17.0 ± 3.7 (7.9–24.4)	0.053	63.8 ± 14.0 (35.0–95.6)	64.7 ± 13.5 (35.3–93.8)	0.420
Affected eye DLDP^a^	21.4 ± 5.4 (14.0–32.2)	19.2 ± 4.1 (13.8–31.7)	0.020	56.9 ± 11.7 (39.4–79.5)	57.9 ± 12.1 (40.8–79.5)	0.041
Unaffected eye DLDP^a^	18.6 ± 5.1 (10.8–30.4)	19.6 ± 2.9 (14.8–24.0)	0.204	56.0 ± 15.3 (37.3–83.2)	55.6 ± 15.1 (35.5–83.0)	0.052

*DLDP* dependent lateral decubitus position, *IOP* intraocular pressure; OPP, ocular perfusion pressure

T1, sitting position; T2, 10 min after supine position; T3, 10 min after right lateral decubitus position; T4, 10 min after supine position; T5, 10 min after left lateral decubitus position; T6, 10 min after supine position

Data are described as the mean ± standard deviation (minimum–maximum)

^*^ Wilcoxon signed-rank test

^a^The DLDP was defined as the position when the eye of interest (affected or unaffected) was placed on the dependent (lower) side in lateral decubitus position

Interestingly, of the 17 patients who demonstrated DLDP-induced IOP elevation, three showed an IOP increase of more than 10 mmHg from the supine position, and the IOPs of the affected eyes exceeded 30 mmHg (31.7 mmHg, 32.2 mmHg, and 30.1 mmHg; z-score 2.05, 1.77, 2.14, respectively) in the DLDP. The corresponding values for unaffected eyes were 17.5 mmHg, 18.8 mmHg, and 18.0 mmHg; z-score − 0.30, −0.01, and 2.47, respectively. In addition, these patients showed an OPP reduction of more than 20 mmHg in the affected eyes after changing from the supine position to the DLDP. Out of the 20 patients, 17 (85%) exhibited IOP elevations and 20 (100%) showed OPP reductions in affected eyes when body posture was changed from the supine to DLPD. However, only 12 (60%) of the 20 patients showed DLDP-induced IOP elevation and OPP reduction in unaffected eyes. More patients exhibited an OPP reduction in the affected eyes compared with the unaffected eyes (*P* = 0.002), and there was a trend for more patients exhibiting IOP elevation in the affected eyes compared with the unaffected eyes, although this was not significant (*P* = 0.074).

Table 3 shows the alterations in IOP and OPP during body position changes. Inter-eye comparison revealed no statistically significant differences at any time point. However, a significant increase in IOP was observed in the affected eyes compared with the unaffected eyes when patients changed their posture from supine to DLDP (+2.9 ± 4.4 mmHg versus +0.7 ± 3.1 mmHg, respectively; *P* = 0.003). The reverse was observed when the patients changed from the DLDP to the supine position; there was a significant decrease in affected eyes compared with unaffected eyes (−3.7 ± 3.2 mmHg versus −1.0 ± 3.3 mmHg, respectively, *P* < 0.001). Similarly, although the inter-eye comparisons showed no statistical differences at any time point, a significant reduction in OPP was observed in affected eyes compared with unaffected eyes when patients changed from supine position to DLDP (−6.7 ± 9.4 mmHg versus −4.9 ± 8.0 mmHg, respectively; *P* = 0.022). Moreover, a significant increase in OPP was observed after changing from DLDP to supine position (+7.8 ± 9.4 mmHg versus +6.2 ± 9.6 mmHg, respectively; *P* = 0.005). To minimize the effect of BP difference between right and left LDP, the changes of IOP and OPP in the right eye- and left eye-affected patients were analyzed separately. The right eye-affected patients showed significant IOP fluctuations in right (affected) eyes compared to left eyes in the postural changes to and from right LDP (Additional file 2). No significant differences were observed in the postural changes to and from left LDP in the right eye-affected patients. In addition, apparent reduction in OPP was observed in the right eyes when changing position from supine to right LDP. Interestingly, left eye-affected patients showed similar patterns in changes of IOP and OPP compared to those shown in right eye-affected patients (Additional file 3).

**Table 3:** Alterations in Intraocular Pressure and Ocular Perfusion Pressure During Changing Body Positions

	Alterations in IOP (mmHg)			Alterations in OPP (mmHg)		
	Affected eye	Unaffected eye	*P* value^*^	Affected eye	Unaffected eye	*P* value^*^
T1 to T2	1.3 ± 2.7	1.8 ± 2.2	0.422	15.6 ± 5.8	15.1 ± 4.6	0.411
T2 to T3	2.4 ± 3.4	1.9 ± 3.1	0.376	−5.9 ± 8.9	−5.9 ± 8.1	0.717
T3 to T4	−2.0 ± 3.5	−1.5 ± 3.4	0.601	5.0 ± 11.0	5.5 ± 9.8	0.823
T4 to T5	2.1 ± 4.6	1.2 ± 3.4	0.936	−11.6 ± 8.2	−11.2 ± 7.7	0.936
T5 to T6	−2.3 ± 3.7	−1.8 ± 2.4	0.896	10.7 ± 9.5	10.2 ± 10.9	0.794
Supine to DLDP^a^	2.9 ± 4.4	0.7 ± 3.1	0.003	−6.7 ± 9.4	−4.9 ± 8.0	0.022
DLDP to supine^b^	−3.7 ± 3.2	−1.0 ± 3.3	<0.001	7.8 ± 9.4	6.2 ± 9.6	0.005

*IOP* intraocular pressure, *OPP* ocular perfusion pressure; T1, sitting position; T2, 10 min after supine position; T3, 10 min after right lateral decubitus position; T4, 10 min after supine position; T5, 10 min after left lateral decubitus position; T6, 10 min after supine position

Data are described as the mean ± standard deviation

The DLDP was defined as the position when the eye of interest (affected or unaffected) was placed on the dependent (lower) side in lateral decubitus position

^*^ Wilcoxon signed-rank test, ^a^ T2 to T3 for right eye and T4 toT5 for left eye, ^b^ T3 to T4 for right eye and T5 to T6 for left eye

**Fig. 1:**
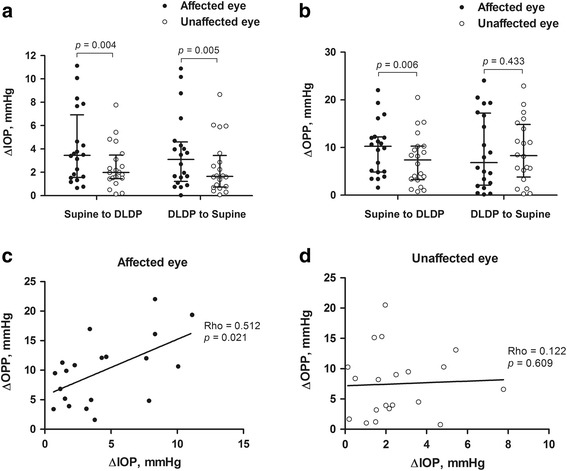
Absolute changes in IOP and OPP values in both eyes of patients with NAION. **a** Scatterplot with median and interquartile range of individual data showing absolute changes in IOP during position change from supine position to dependent lateral decubitus position (DLDP). **b** Scatterplot with median and interquartile range of individual data showing absolute changes in OPP during position change from supine position to DLDP. **c** Scatterplot with regression curve showing the significant positive association between absolute IOP changes and OPP changes during position change from supine to DLDP in affected eyes. **d** Scatterplot with regression curve showing no significant association between IOP changes and OPP changes during position change from supine to DLDP in unaffected eyes

The absolute changes of IOP and OPP are shown in Fig. 1. Compared to unaffected eyes, affected eyes showed significantly larger absolute changes in IOP (4.13 ± 3.19 mmHg versus 2.51 ± 1.92 mmHg, respectively; *P* = 0.004) and OPP (9.86 ± 5.69 mmHg versus 7.50 ± 5.49 mmHg, respectively; *P* = 0.005) when changing position from supine to DLDP. In addition, changing from DLDP back to supine position resulted in greater changes in IOP in affected eye compared with unaffected eye (3.68 ± 3.18 mmHg versus 2.48 ± 2.37 mmHg, respectively; *P* = 0.006), but the changes in OPP were not significant (9.24 ± 7.89 mmHg versus 9.17 ± 6.64 mmHg, respectively; *P* = 0.433). Affected eye showed significant positive correlation between absolute change in IOP and OPP when changing position from supine to DLDP (Rho = 0.512, *P* = 0.021); however, the correlation was not significant in unaffected eye (Rho = 0.122, *P* = 0.609). The individual data for this study is available as Additional file 4.

## Discussion

NAION is presumed to be caused by acute hemodynamic instability in optic nerve circulation, resulting in ONH infarction. It is important to understand that ONH perfusion is dependent on the balance between MAP and IOP, meaning that a significant increase in IOP, a reduction in MAP, or both, can contribute to an increased incidence of NAION [1–3]. An acute increase in IOP secondary to other causes can induce ONH hypoperfusion that may result in NAION, as evidenced by previous case reports of cataract extractions or acute angle closure glaucoma [16, 17]. In addition, a reduction in MAP is another postulated mechanism to explain NAION that has been widely reported in cases of acute blood loss during surgery, shock, and hemodialysis [18, 19].

It is important to note that NAION develops in most patients during sleep. It has been reported that at least 73.3% of patients discovered their visual loss on awakening or early after awakening, further indicating that development of NAION is closely related to sleep [3, 9, 10, 20]. Therefore, focusing attention onto what occurs during sleeping hours may improve our understanding of the pathophysiology of NAION. During sleep, arterial blood pressure decreases due to the attenuation of the sympathetic tone; this nocturnal hypotension is widely accepted as a critical predisposing factor for the development of NAION in patients with vulnerable ONHs [9, 10, 20]. In addition, during sleep, people may adopt various body postures. According to previous reports, IOP can become significantly elevated following postural changes from the sitting to the supine or LDP, due to increases in episcleral venous pressure or alterations in the rate of uveoscleral outflow [6, 21, 22]. These postural changes during sleep can significantly alter IOP that may in turn further deteriorate the perfusion status of ONHs already susceptible to ischemia.

In the present study, the extent of posture-induced IOP variation was greater in affected eyes compared with unaffected eyes, particularly when patients changed their position from supine to DLDP. De Koninck et al., [23] in a study on sleep position and shifting, showed that with advanced age, sleep position patterns change with increased preference for the side sleep position, fewer position changes during the night, and increased amounts of time spent in one position. In the present study, when changing positions from sitting to supine, IOP was significantly elevated in both the affected and unaffected eyes, however, inter-eye differences were not statistically significant. This is in concordance with a previous study by James et al. [11] in which IOP was demonstrated to be significantly elevated in both NAION affected eyes (3.9 mmHg) and unaffected eyes (3.6 mmHg); however, differences between these groups of eyes were minimal (0.3 mmHg).

Although the IOP of affected eyes increased in the right or left LDP compared with the supine position (2.4 mmHg and 2.1 mmHg, respectively), the magnitude was greater when the affected eye was on the dependent side in the LDP (2.9 mmHg). The extent of IOP elevation in the NAION-affected eyes after changing from the supine to DLDP was greater than that reported in previous studies of glaucoma patients (worse eye, 2.3 mmHg) [5, 6]. Such IOP elevations induced by sleeping body posture, combined with nocturnal arterial hypotension, may trigger acute circulatory disturbances in at-risk ONHs [2, 3, 11]. These findings implicate significant IOP elevation in certain sleeping body positions as one of the predisposing factors for NAION development.

Previous studies have postulated that the right-left cardiovascular hemodynamics in LDP could be different due to the anatomy of the left-sided heart [24, 25]. Accordingly, the right eye- and left eye-affected patients were analyzed separately. The results for separate analysis were similar; affected eyes showed significant fluctuations of IOP and OPP during changing position from supine to affected eye DLDP. Therefore, minor differences of blood pressure in the right and left LDP could not disturb our conclusion, although we could not completely exclude the effect of blood pressure difference on the results in this study. The evaluation of arterial pressure on the OPP was beyond the scope of our study, although future investigation should be carried out. One interesting finding is that some of the left eye-affected patients showed significant IOP and OPP changes in the right unaffected eyes during positional changes from supine to right LDP (Additional file 3). Close follow up of the unaffected eyes of these patients should be warranted to find out whether NAION occurs in the future.

Interestingly, our data showed that in addition to the significant changes in IOP and OPP, a significant correlation between these two values was noted especially when the patients changed their position from supine to DLDP. These findings implicate that during sleeping, postural changes to DLDP could significantly alter OPP, which in turn could make the eye prone to developing NAION. In addition, when posing DLDP, the eye in the lower position can be easily compressed by the pillow or one’s arm which might result in significant IOP fluctuation of the eye. Precautions should be taken for people with risk factors of NAION who prefer DLDP and frequently changes to this position during their sleeping.

Although IOP and OPP were the primary focus of our study, it is important to recognize systemic risk factors that predispose patients to ischemia, including arterial hypertension (25%), diabetes (20%), hyperlipidemia (10%), thyroid disease (15%), arteriosclerosis with ischemic heart disease (10%), and cerebrovascular diseases (25%). These risk factors may disrupt the vascular autoregulation process of arterial flow as a result of vasomotor dysfunction of endothelial cells, predisposing the eye to ischemic damage [2, 26]. Our patients may also have had an increased sensitivity to minor changes in IOP, biasing them toward developing ONH ischemia.

In addition to the small sample size, wide range of age, and the absence of a control group, our study has several limitations. First, the study was not performed during actual sleeping time. Consequently, our findings may not represent actual variations in IOP that may differ due to the various physiological and environmental changes that occur during sleep [13, 27]. However, our study was designed to specifically assess the impact of postural changes on IOP and OPP that may arise during sleep [7]. In addition, considering that IOP is higher during sleeping than waking hours, our findings suggest that, combined with nocturnal arterial hypotension, posture-induced IOP elevated above normal levels may be more critical to the development of ischemia in a vulnerable ONH when sleeping [1–3]. Second, the accuracy of IOP measurements by rebound tonometry may be debated. However, the accuracy of Icare has been shown to be comparable to that of Goldmann applanation tonometry [28]. Third, OPP was calculated according to theoretical formulas; therefore, it may not reflect the actual physiological ocular perfusion status. Direct measurements of ocular blood flow may have produced different outcomes. Currently, there is no single reliable clinical method that measures ONH blood flow [2, 29]. Further studies incorporating direct measurements of actual blood flow in the ONH are warranted. Fourth, IOP and OPP changes in prone position, with the head turned to specific sides, were not evaluated. Such changes may have significantly affected the ONH blood flow [12].

## Conclusion

In summary, our study demonstrated that there are significant IOP fluctuations in affected eyes compared with unaffected eyes in the postural change to and from dependent LDP. Furthermore, a reduction in OPP was apparent in affected eyes when patients changed their body posture from supine to DLDP. These findings support the concept that posture-induced IOP changes may be an important predisposing factor for NAION development. We believe that these findings contribute to our understanding of the mechanisms underlying NAION pathogenesis.

## Additional files

Additional file 1: **Table S1.** Blood pressure at each time point after changing body posture. (DOCX 16 kb)

Additional file 2: **Table S2.** Alterations in intraocular pressure and ocular perfusion pressure during changing body positions in right eye-affected patients. (DOCX 18 kb)

Additional file 3: **Table S3.** Alterations in intraocular pressure and ocular perfusion pressure during changing body positions in left eye-affected patients. (DOCX 18 kb)

Additional file 4: Individual data of blood pressure, intraocular pressure, and ocular perfusion pressure on each time point. **Figure S1.** Individual blood pressure data at each time point. **Figure S2.** Individual intraocular pressure data at each time point. **Figure S3.** Individual ocular perfusion pressure data at each time point. **Figure S4.** Individual data of alterations in intraocular pressure during changing body position. **Figure S5.** Individual data of alterations in ocular perfusion pressure during changing body position. (DOCX 1701 kb)

## Abbreviations

BP: Blood pressure
DLDP: Dependent LDP
IOP: Intraocular pressure
LDP: Lateral decubitus position
MAP: Mean arterial pressure
NAION: Non-arteritic anterior ischemic optic neuropathy
ONH: Optic nerve head
OPP: Ocular perfusion pressure
