# Supplementary material for: Postural effects on intraocular pressure and ocular perfusion pressure in patients with non-arteritic anterior ischemic optic neuropathy
Source: BMC Ophthalmol. 2017 Apr 20;17:47. doi: 10.1186/s12886-017-0441-3 (PMC5397825; doi:10.1186/s12886-017-0441-3)
Supplement: Supplementary file 1 — Table S1. Blood pressure at each time point after changing body posture. (DOCX 16 kb) [file 12886_2017_441_MOESM1_ESM.docx]

Additional file 1. Table S1. Blood pressure at each time point after changing body posture

| Time point | Position | SBP  (mmHg) | DBP  (mmHg) | Mean BP (mmHg)* |
| --- | --- | --- | --- | --- |
| T1 | Sitting | 130.00 ± 20.11 | 82.35 ± 14.44 | 98.23 ± 15.86 |
| T2 | 10 min after supine | 125.50 ± 18.61 | 78.85 ± 13.40 | 94.40 ± 14.91 |
| T3 | 10 min after right LDP | 122.60 ± 23.55 | 73.90 ± 15.62 | 90.13 ± 17.80 |
| T4 | 10 min after supine | 126.20 ± 19.94 | 78.00 ± 12.67 | 94.07 ± 14.61 |
| T5 | 10 min after left LDP | 116.15 ± 23.00 | 66.40 ± 13.67 | 82.98 ± 16.38 |
| T6 | 10 min after supine | 125.65 ± 23.42 | 75.80 ± 13.52 | 92.42 ± 16.14 |

BP = blood pressure; DBP = diastolic blood pressure; LDP = lateral decubitus position; SBP = systolic blood pressure

Data are described as the mean ± standard deviation

^*^Mean BP = 2/3 DBP + 1/3 SBP
