# Supplementary material for: Postural effects on intraocular pressure and ocular perfusion pressure in patients with non-arteritic anterior ischemic optic neuropathy
Source: BMC Ophthalmol. 2017 Apr 20;17:47. doi: 10.1186/s12886-017-0441-3 (PMC5397825; doi:10.1186/s12886-017-0441-3)
Supplement: Supplementary file 2 — Table S2. Alterations in intraocular pressure and ocular perfusion pressure during changing body positions in right eye-affected patients. (DOCX 18 kb) [file 12886_2017_441_MOESM2_ESM.docx]

**Additional file 2. Table S2. Alterations in Intraocular Pressure and Ocular Perfusion Pressure During Changing Body Positions** **in a right eye-affected patients**

|  | **Alterations in IOP (mmHg)** | |  | **Alterations in OPP (mmHg)** | |  |
| --- | --- | --- | --- | --- | --- | --- |
|  | Affected eye | Unaffected eye | *P* value^*^ | Affected eye | Unaffected eye | *P* value^*^ |
| T1 to T2 | 1.5 ± 0.8 | 2.0 ± 2.0 | 0.396 | 15.1 ± 5.6 | 14.6 ± 4.6 | 0.576 |
| T2 to T3 | 3.0 ± 1.2 | 1.2 ± 2.9 | 0.010 | -7.0 ± 9.6 | -5.5 ± 8.8 | 0.034 |
| T3 to T4 | -3.5 ± 2.8 | -1.1 ± 3.6 | 0.014 | 8.8 ± 9.6 | 8.1 ± 9.6 | 0.506 |
| T4 to T5 | 1.8 ± 3.5 | 1.6 ± 3.2 | 0.900 | -14.6 ± 5.6 | -15.2 ± 4.8 | 0.440 |
| T5 to T6 | -1.4 ± 3.3 | -2.4 ± 1.8 | 0.382 | 13.2 ± 8.9 | 14.2 ± 9.7 | 0.357 |

IOP, intraocular pressure; OPP, ocular perfusion pressure; T1, sitting position; T2, 10 min after supine position; T3, 10 min after right lateral decubitus position; T4, 10 min after supine position; T5, 10 min after left lateral decubitus position; T6, 10 min after supine position.

Data are described as the mean ± standard deviation.

^*^Wilcoxon signed-rank test.
