# Supplementary material for: Postural effects on intraocular pressure and ocular perfusion pressure in patients with non-arteritic anterior ischemic optic neuropathy
Source: BMC Ophthalmol. 2017 Apr 20;17:47. doi: 10.1186/s12886-017-0441-3 (PMC5397825; doi:10.1186/s12886-017-0441-3)
Supplement: Supplementary file 3 — Table S3. Alterations in intraocular pressure and ocular perfusion pressure during changing body positions in left eye-affected patients. (DOCX 18 kb) [file 12886_2017_441_MOESM3_ESM.docx]

**Additional file 3. Table S3. Alterations in Intraocular Pressure and Ocular Perfusion Pressure During Changing Body Positions in a left eye-affected patients**

|  | **Alterations in IOP (mmHg)** | |  | **Alterations in OPP (mmHg)** | |  |
| --- | --- | --- | --- | --- | --- | --- |
|  | Affected eye | Unaffected eye | *P* value^*^ | Affected eye | Unaffected eye | *P* value^*^ |
| T1 to T2 | 0.8 ± 3.0 | 1.4 ± 2.7 | 0.499 | 16.5 ± 6.4 | 15.9 ± 5.0 | 0.582 |
| T2 to T3 | 1.2 ± 3.7 | 3.2 ± 2.6 | 0.088 | -4.0 ± 7.6 | -6.6 ± 7.2 | 0.018 |
| T3 to T4 | -0.7 ± 3.0 | -2.2 ± 3.0 | 0.249 | 2.1 ± 10.2 | 0.8 ± 8.8 | 0.236 |
| T4 to T5 | 2.6 ± 6.5 | 0.3 ± 3.8 | 0.029 | -6.1 ± 9.7 | -3.9 ± 6.8 | 0.018 |
| T5 to T6 | -4.1 ± 3.9 | -0.8 ± 3.0 | 0.003 | 6.0 ± 9.35 | 2.7 ± 9.3 | 0.003 |

IOP, intraocular pressure; OPP, ocular perfusion pressure; T1, sitting position; T2, 10 min after supine position; T3, 10 min after right lateral decubitus position; T4, 10 min after supine position; T5, 10 min after left lateral decubitus position; T6, 10 min after supine position.

Data are described as the mean ± standard deviation.

^*^Wilcoxon signed-rank test.
