# Supplementary material for: Postural effects on intraocular pressure and ocular perfusion pressure in patients with non-arteritic anterior ischemic optic neuropathy
Source: BMC Ophthalmol. 2017 Apr 20;17:47. doi: 10.1186/s12886-017-0441-3 (PMC5397825; doi:10.1186/s12886-017-0441-3)
Supplement: Supplementary file 4 — Individual data of blood pressure, intraocular pressure, and ocular perfusion pressure on each time point. Figure S1. Individual blood pressure data at each time point. Figure S2. Individual intraocular pressure data at each time point. Figure S3. Individual ocular perfusion pressure data at each time point. Figure S4. Individual data of alterations in intraocular pressure during changing body position. Figure S5. Individual data of alterations in ocular perfusion pressure during changing body position. (DOCX 1701 kb) [file 12886_2017_441_MOESM4_ESM.docx]

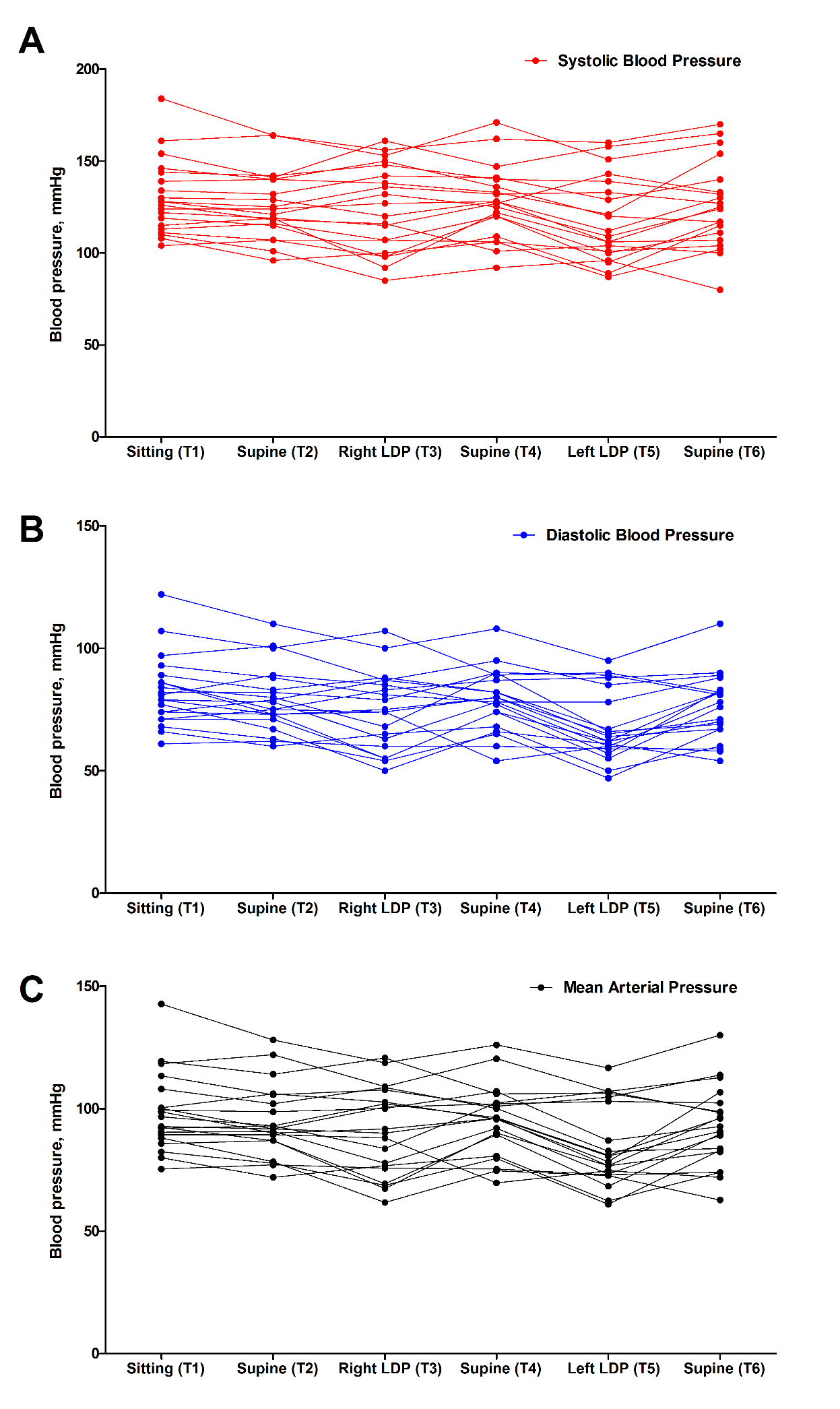
Figure S1. Individual blood pressure data at each time point. (A) Systolic blood pressure. (B) Diastolic blood pressure. (C) Mean arterial pressure.


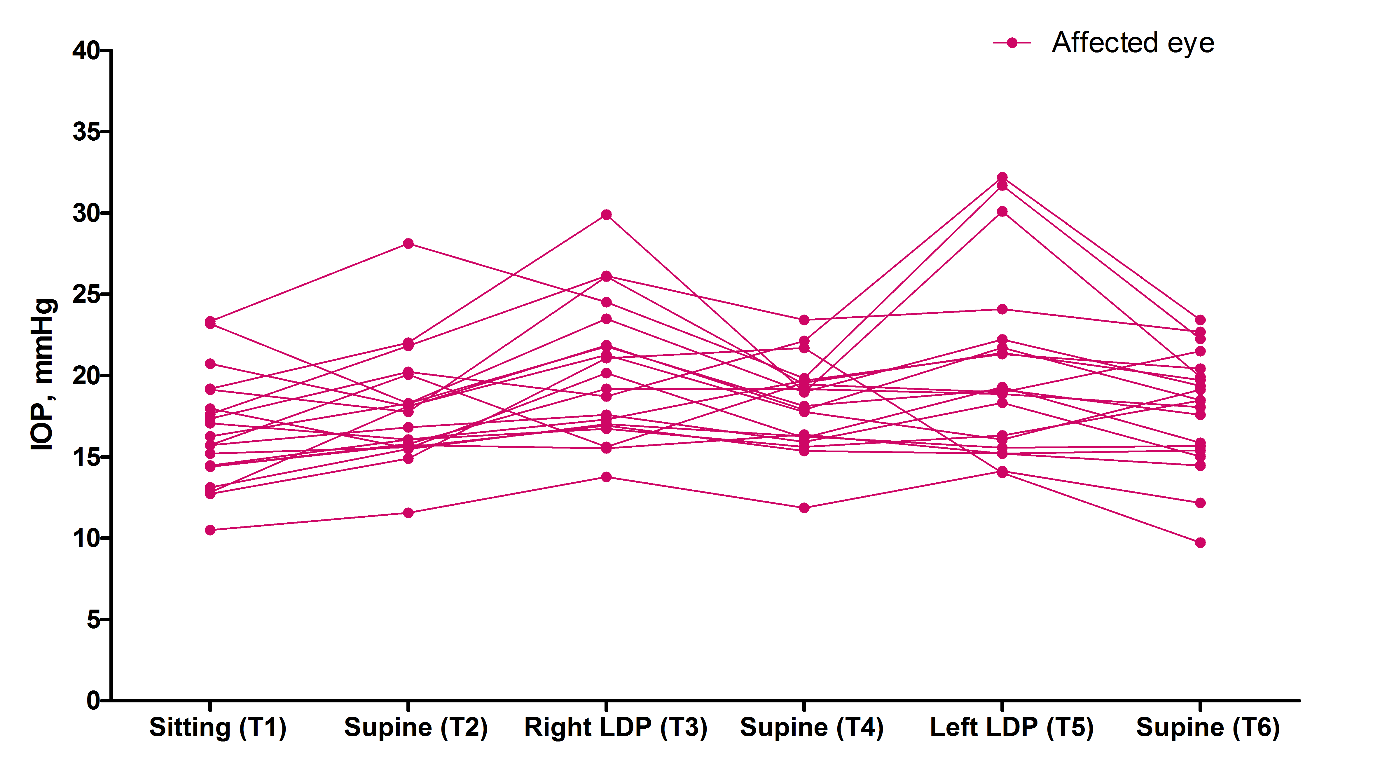

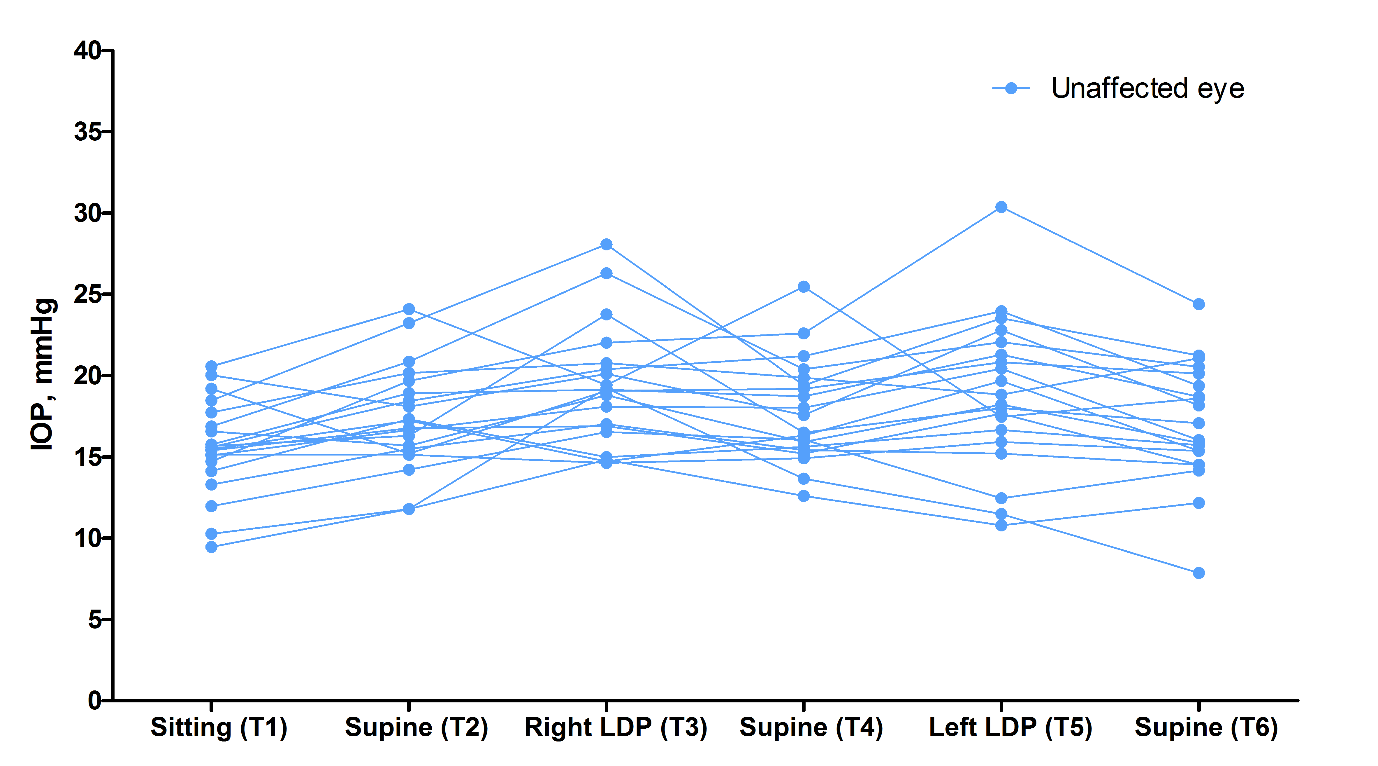


Figure S2. Individual intraocular pressure (IOP) data at each time point.


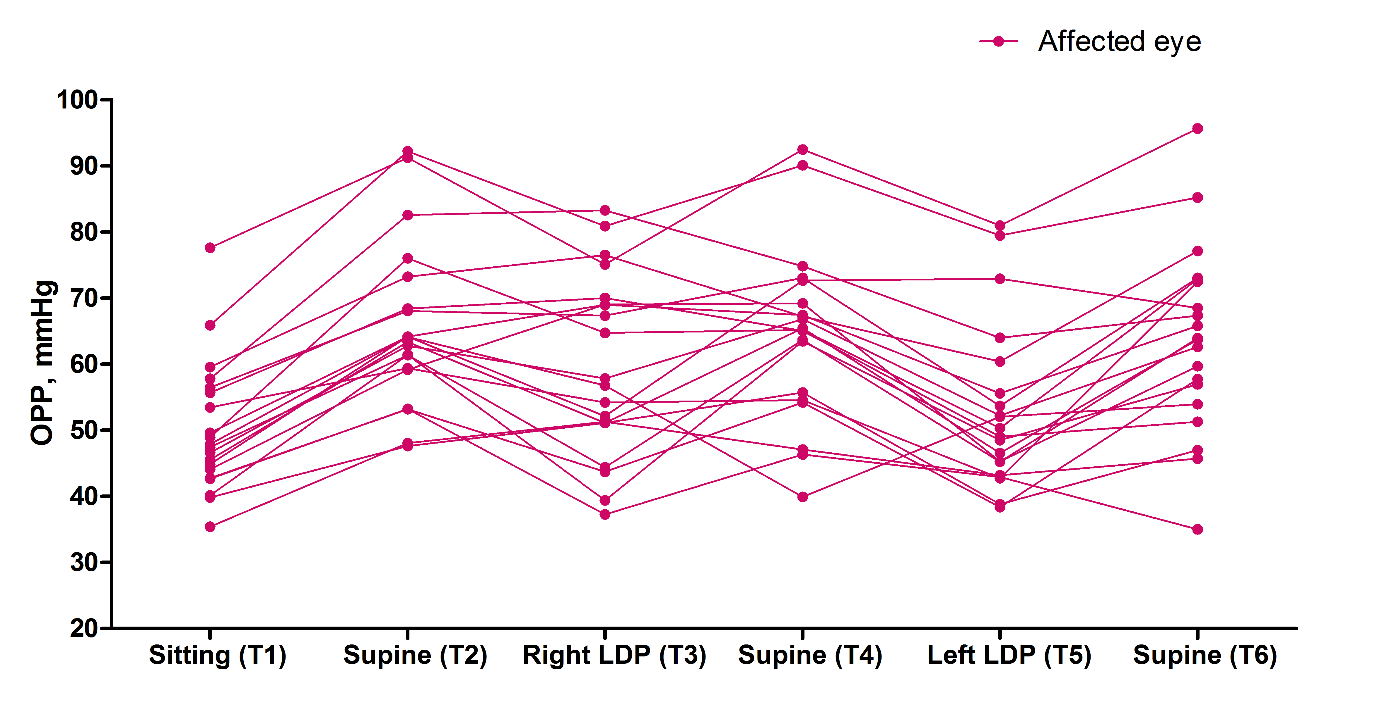

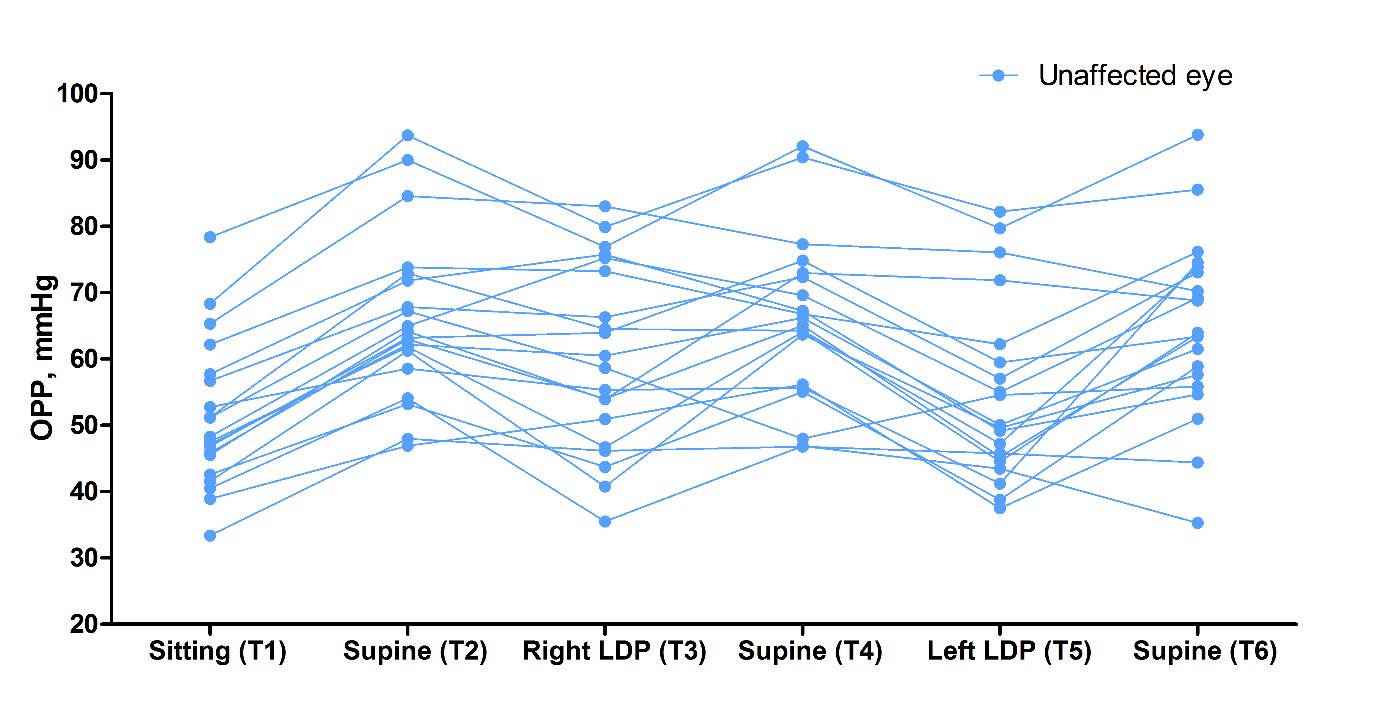


Figure S3. Individual ocular perfusion pressure (OPP) data at each time point.


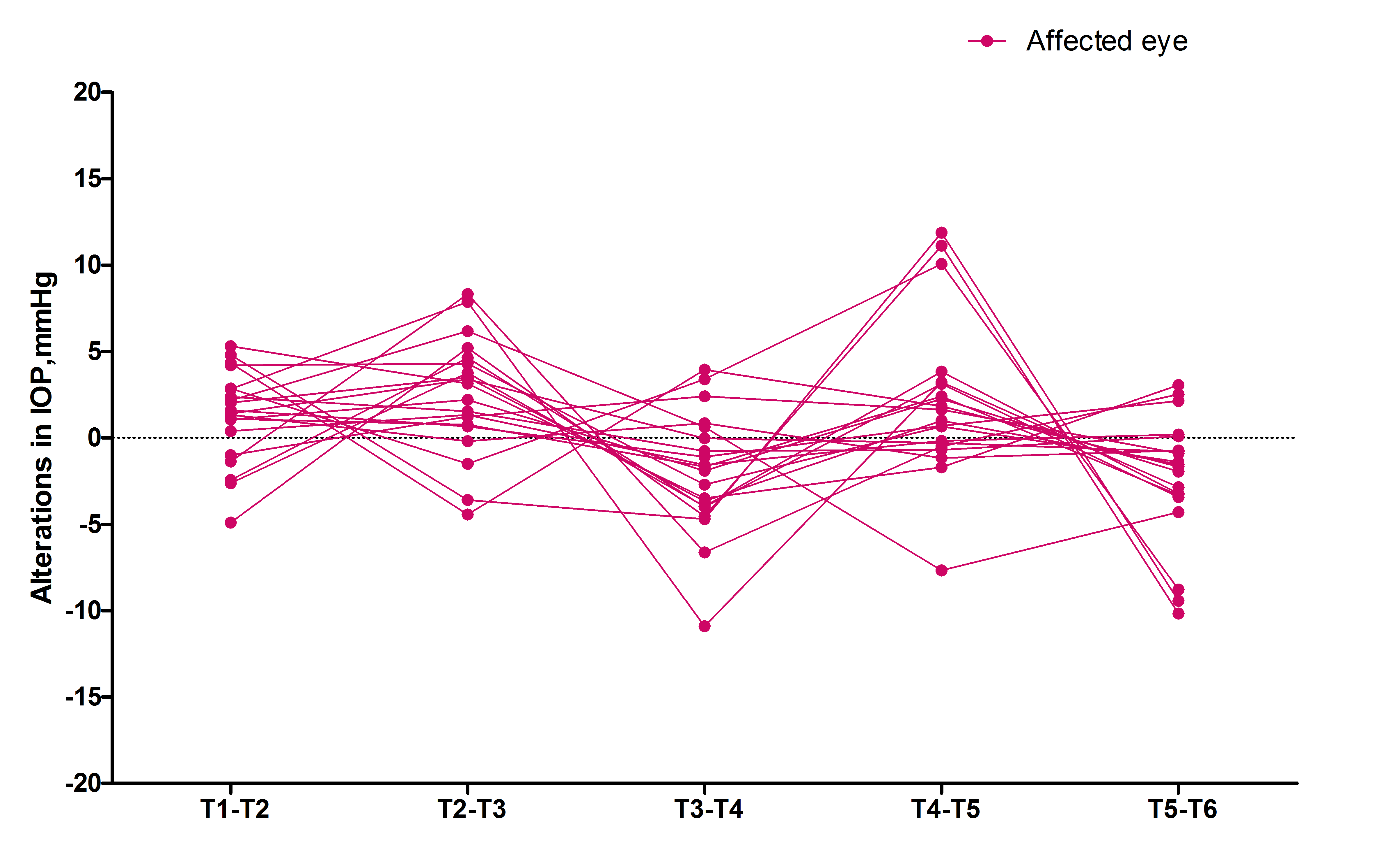


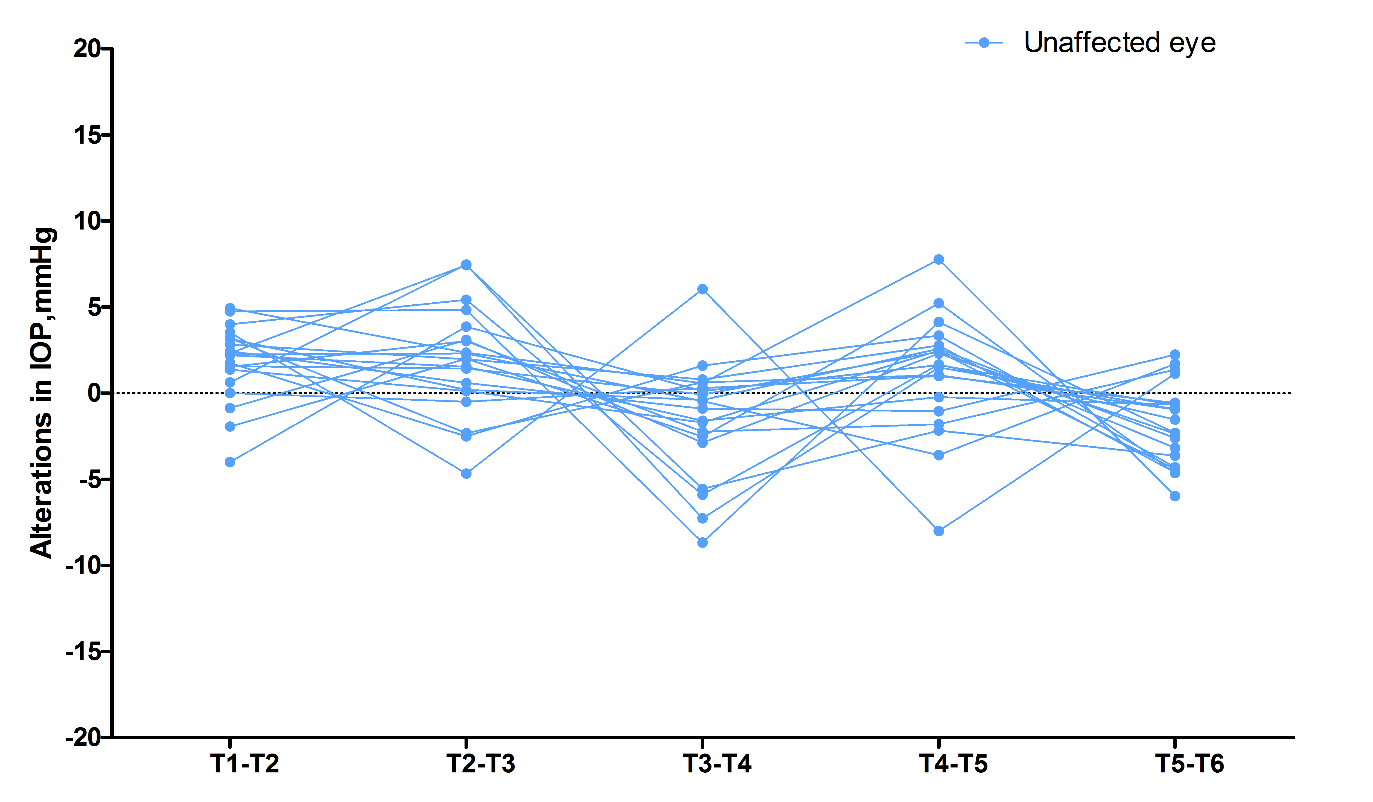


Figure S4. Individual data of alterations in intraocular pressure (IOP) during changing body position.


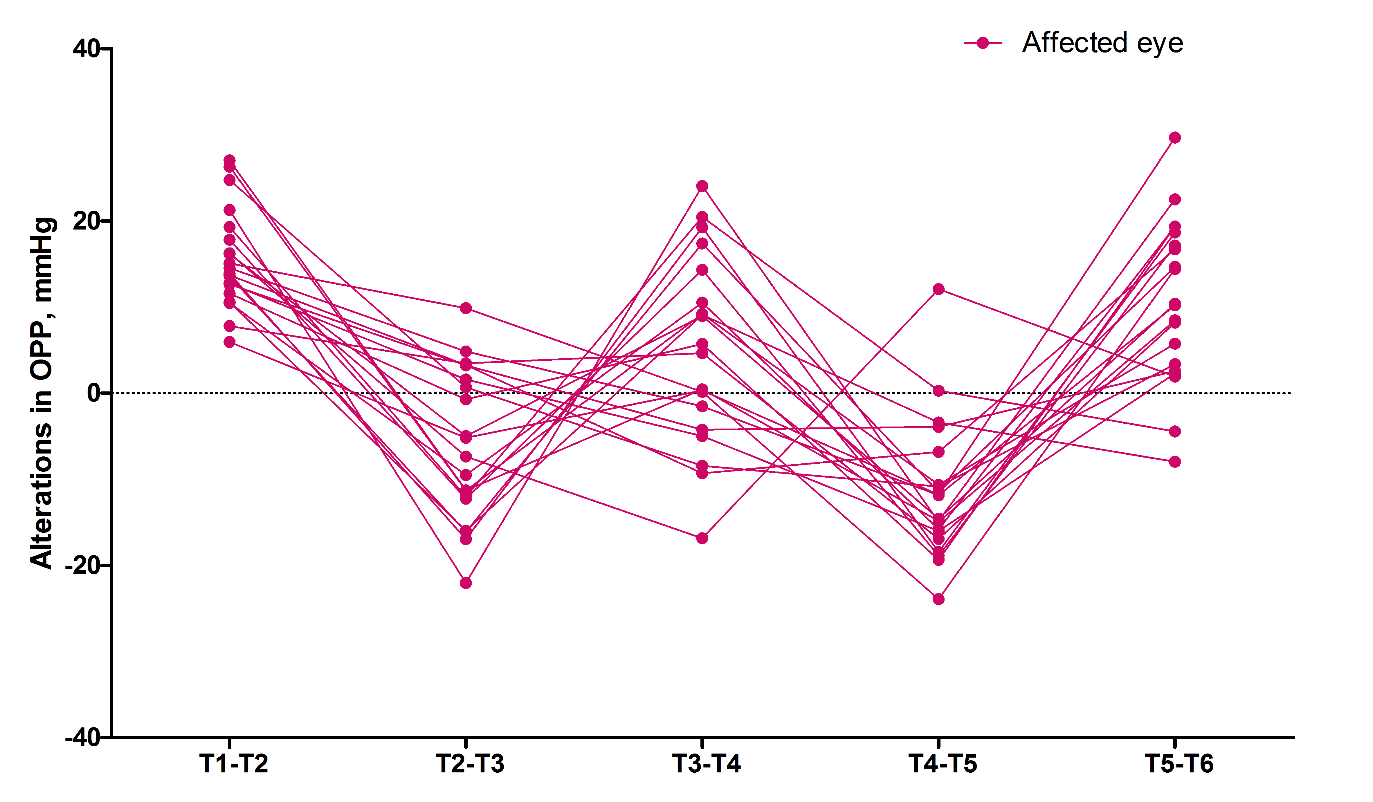


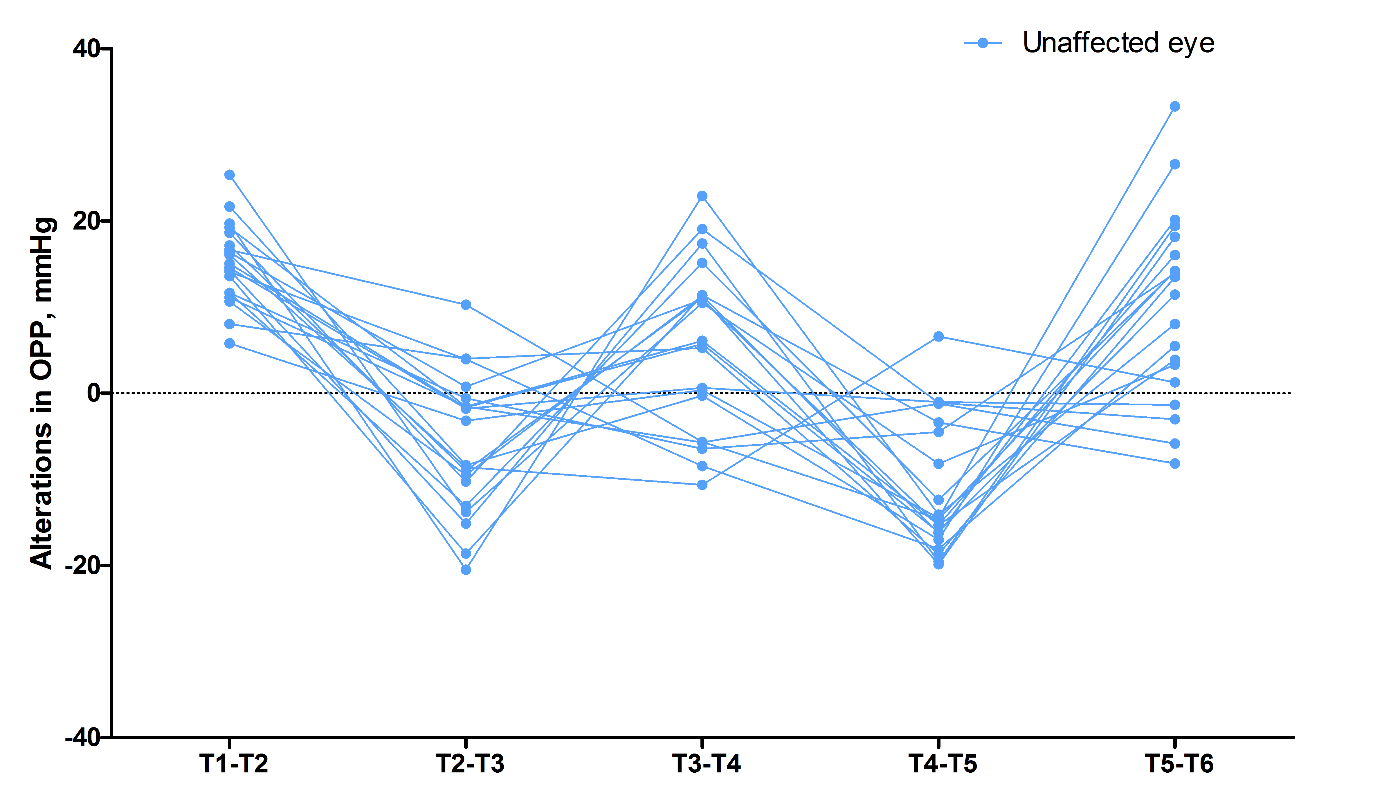


Figure S5. Individual data of alterations in ocular perfusion pressure (OPP) during changing body position.
